# Long-term efficacy and safety of E/C/F/TDF vs EFV/FTC/TDF and ATV+RTV+FTC/TDF in HIV-1-infected treatment-naïve subjects ≥50 years

**DOI:** 10.7448/IAS.17.4.19767

**Published:** 2014-11-02

**Authors:** Brian Gazzard, Pierre Marie Girard, Giovanni Di Perri, Joel Gallant, William Towner, Felipe Rogatto, Jennifer Demorin, Damian McColl, Hui Liu, Martin Rhee, Javier Szwarcberg, David Piontkowsky

**Affiliations:** 1 Infectious Disease, Chelsea and Westminster Hospital, London, UK; 2 Infectious Diseases, Center Hospital at University St Antoine, Paris, France; 3 Internal Medicine, School of Medicine, University of Turin, Torino, Italy; 4 Infectious Diseases, Southwest CARE Center, Santa Fe, NM, USA; 5 Internal Medicine, Kaiser Permanente, Los Angeles, CA, USA; 6 Medical Affairs, Gilead Sciences Europe, Stockley Park, UK; 7 HIV Medical Affairs, Gilead Sciences, Foster City, CA, USA; 8 Biostats, Gilead Sciences, Foster City, CA, USA; 9 Clinical Research, Gilead Sciences, Foster City, CA, USA

## Abstract

**Introduction:**

In high-income countries, ≥30% of HIV-infected patients are ≥50 years (yrs) old (UNAIDS 2013). In two phases, three clinical trials (Studies 102 and 103) elvitegravir/cobicistat/emtricitabine/tenofovir DF (E/C/F/TDF; STB) had non-inferior efficacy and favourable safety vs efavirenz/emtricitabine/tenofovir DF (EFV/FTC/TDF; ATR) or ritonavir-boosted atazanavir (ATV+RTV)+FTC/TDF (TVD) in HIV-infected, treatment-naïve subjects at Week 144. The efficacy and safety of STB in subjects < or ≥50 yrs is described.

**Materials and Methods:**

Post hoc analysis of efficacy, tolerability and safety in subjects < or ≥50 yrs at Week 144.

**Results:**

Subjects ≥50 yrs in Study 102: STB: 14% (49/348), ATR: 16% (56/352); in Study 103: STB: 14% (48/353), ATV+RTV+TVD: 14% (48/355). Efficacy, safety and tolerability by age and study endpoint are shown in Table 1. Regardless of age, STB had robust efficacy at Week 144 with similar virologic outcomes vs ATR or ATV+RTV+TVD. Discontinuations (DC) due to AE on STB were similar to the comparators, most occurred by Week 48. Median changes in eGFR on STB were similar by age; DC with renal PRT was rare [STB: 4 (0.6%); ATV: 3 (0.8%); ATR: 0], 2 and 1 in ≥50 yrs old strata, respectively.

**Conclusions:**

STB compared to ATR or ATV+RTV+TVD, is an efficacious, well-tolerated and safe regimen for HIV-1-infected, treatment-naïve subjects<or≥50 yrs of age.

**Table 1:** Efficacy, safety and tolerability by age

n (%)	Week 48<50	Week 48≥50	Week 96<50	Week 96≥50	Week 144<50	Week 144≥50
STB versus ATR						
Virologic success (VS)	263 (88); 250 (84)	42 (86); 46 (82)	253 (85); 241 (81)	40 (82); 46 (82)	241 (81); 221 (75)	38 (78); 44 (79)
Virologic failure (VF)	23 (8); 23 (8)	2 (4); 2 (4)	20 (7); 24 (8)	2 (4); 3 (5)	23 (8); 30 (10)	3 (6); 4 (7)
Mean change in CD4, cells/mm^3^	246; 208	199; 194	305; 278	233; 250	325; 304	295; 280
AEs Leading to DC	10 (3); 12 (4)	3 (6); 6 (11)	12 (4); 18 (6)	5 (10); 6 (11)	16 (5); 20 (7)	5 (10); 6 (11)
Median change in eGFRCG, mL/min	−14.8; −1.6	−12.0; −7.5	−13.9; 0.4	−12.9; −8.2	−15.2; −0.3	−18.2; −8.7
STB versus ATV+RTV+TVD						
VS	271 (89); 266 (87)	45 (94); 42 (88)	251 (82); 249 (81)	43 (90); 43 (90)	236 (77); 228 (74)	38 (79); 37 (77)
VF	17 (6); 17 (6)	2 (4); 2 (4)	22 (7); 25 (8)	2 (4); 1 (2)	25 (8); 25 (8)	3 (6); 1 (2)
Mean change in CD4, cells/mm^3^	212; 213	176; 200	261; 266	226; 231	285; 300	245; 256
AEs Leading to DC	12 (4); 15 (5)	1 (2); 3 (6)	13 (4); 17 (6)	2 (4); 4 (8)	17 (6); 21 (7)	4 (8); 9 (19)
Median change in eGFRCG, mL/min	−13.4; −10.0	−10.2; −7.3	−12.5; −8.8	−11.1; −12.6	−12.9; −9.4	−13.5; −12.9

